# Targeted temperature management after out-of-hospital cardiac arrest: certainties and uncertainties

**DOI:** 10.1186/s13054-014-0459-3

**Published:** 2014-07-22

**Authors:** Matt P Wise, Janneke Horn, Anders Åneman, Niklas Nielsen

**Affiliations:** Adult Critical Care, University Hospital of Wales, Heath Park, Cardiff, CF14 4XW UK; Department of Intensive Care, Academic Medical Center, Postbus 22700 NL-1100 DE, Amsterdam, The Netherlands; Department of Intensive Care, Liverpool Hospital, Locked Bag 7103, Liverpool, BC NSW 1871 Australia; Department of Anesthesiology and Intensive Care, Helsingborg Hospital, S Vallgatan 5, S-25187 Helsingborg, Sweden; Department of Clinical Sciences, Lund University, Box 117, S-22100 Lund, Sweden

Targeted temperature management was adopted as part of the treatment of unconscious survivors of out-of-hospital cardiac arrest following the publication of two landmark studies [1,2] which concluded that mild induced hypothermia (32°C to 34°C) improved survival and neurological outcome, substantiating the neuroprotective effect of mild hypothermia described in experimental animal data [3]. Subsequently, this therapy was recommended by international guidelines [4,5] and became a standard of care. Although both trials [1,2] had exclusion criteria limiting generalizability, mild induced hypothermia was applied to the wider cardiac arrest population.

Until recently [6], despite uncertainties over what represents the optimal target population, temperature, duration of therapy, or rate of rewarming [5], no large randomized clinical trials (RCTs) had been conducted since 2003. A systematic review and meta-analysis performed by using the Grading of Recommendations Assessment, Development, and Evaluation system [7] and trial sequential analysis [8] concluded that the existing quality of evidence was low and that firm evidence in support of induced hypothermia was lacking [9].

To further investigate targeted temperature management, the Target Temperature Management (TTM) after out-of-hospital cardiac arrest trial group was established. As mild induced hypothermia had become ingrained in clinical practice, it was considered infeasible to compare 32°C to 34°C with no temperature control, as failing to control fever would have been unacceptable to many clinicians. The primary objective of the TTM trial [6] was to compare 33°C with 36°C in unconscious survivors of out-of-hospital cardiac arrest for survival for a minimum follow-up of 6 months. A temperature of 36°C was chosen as registry data showed that 36°C was the median temperature of out-of-hospital cardiac arrest patients arriving at the hospital. Furthermore, it was unlikely that, if feedback-controlled cooling devices were used, the temperature in the 36°C group would stray into the febrile range.

The TTM trial sought to be pragmatic, reflecting current clinical practice [10] of applying mild induced hypothermia to the entire out-of-hospital cardiac arrest population. Arrests of a presumed cardiac cause of all rhythms, including witnessed asystole, were included, as neuroprotection should not depend on initial rhythm. The trial protocol and analysis plan were published in advance and attempted to address possible deficiencies of previous trials [10,11]. These included the following: a sample size twice as large as all previous RCTs combined (939 versus 478) adequately powered to detect or reject differences in survival, documentation of coma level at randomization, avoidance of hyperthermia both during and after completion of target temperature management in unconscious patients, standardized neurological assessment/prognostication, and guidance for withdrawal of treatment (adverse events, including infections, bleeding, electrolyte disturbances, seizures, and arrhythmias, were also recorded); a blinded neurological follow-up [10,12] extending beyond crude scales (Cerebral Performance Category and modified Rankin Scale), including tests on quality of life and cognitive function assessing both survivors and relatives in a face-to-face visit in the majority; and a bio-bank of approximately 20,000 blood samples to investigate the role of biomarkers.

The results of the TTM trial were unequivocal, demonstrating no difference in survival or the secondary outcome of combined survival and neurological outcome [6]. An important design feature of the TTM trial, distinguishing it from earlier trials, was the approach to prognostication and withdrawal of care in patients remaining unconscious [13,14]. Prognostication was delayed for 108 hours (unless specific withdrawal criteria were met) and performed by a clinician who was blinded to the intervention and who then recommended withdrawal, continuation, or no further escalation of care.

The robust design of the TTM trial has been recognized by a number of commentators [13,14], including an author of one of the landmark trials [2]. However, others have questioned the validity of the study, raising concerns over the rate of rewarming, temperature separation and swings, and recruitment rates [15]. It has been argued that the rate of rewarming was rapid, abrogating the benefits of cooling to 33°C [15]. The optimal rate of rewarming remains unknown [5] but was undertaken at a maximum speed of 0.5°C per hour in keeping with current guidelines [5]. The actual rewarming rate in the TTM trial was 0.36 ± 0.13°C per hour, which seems comparable to the rate in the Hypothermia after Cardiac Arrest trial [1], and the two temperature arms were clearly well controlled and separated (Figure 1). The TTM trial aimed to reflect current clinical practice, including as large a population as possible to make the results applicable to use by clinicians. On the basis of registries, 80% of patients meeting inclusion criteria in the TTM trial would be eligible for random assignment after exclusion criteria were considered [16]. The 66% screening to inclusion (and 76% random assignment of patients meeting inclusion criteria) represents a considerable effort by participating sites. Although the study ran more than 26 months, it was not until 9 months that 50% of sites were open to recruitment. The median number of patients randomly assigned per site was 21, and sites above the median recruited 80% of all patients.

**Figure 1:**
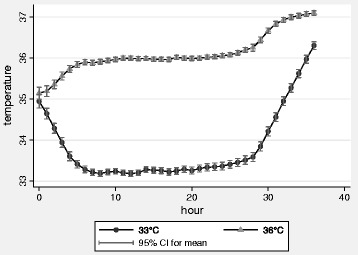
**Mean bladder temperature in the 33°C and 36°C intervention groups of the Target Temperature Management trial, during the 36 hours of temperature intervention.** The temperature graph in the initial publication [6] showed mean body temperature with a dispersion of two standard deviations encompassing 95% of all observations. Temperature values are presented with 95% confidence intervals.

In this trial (the largest one to date), there was no difference in outcomes between unconscious survivors of out-of-hospital cardiac arrest treated at 33°C or 36°C, giving clinicians the option of choosing either temperature management strategy, but the least invasive strategy is compelling [13]. The most interesting aspect of the TTM trial may be that it indicates substantial knowledge gaps in post-cardiac arrest fever and temperature management. The optimal temperature, duration of temperature management, and target population remain to be defined.

## Abbreviations

RCT: Randomized clinical trial
TTM: Target temperature management
